# Mill Scale-Derived Magnetite Nanoparticles: A Novel Substrate for Lactate Oxidase-Based Biosensors

**DOI:** 10.3390/bios13110957

**Published:** 2023-10-27

**Authors:** Hamid Khosravi, Oscar Carreras-Gallo, Jasmina Casals-Terré

**Affiliations:** 1 Department of Mechanical Engineering, Polytechnic University of Catalonia-BarcelonaTech (UPC), 08222 Terrassa, Barcelona, Spain; jasmina.casals@upc.edu; 2 Department of Innovation, Barnasteel S.A., 08755 Castellbisbal, Barcelona, Spain; oscar.carreras2@gcelsa.com

**Keywords:** mill scale, magnetite nanoparticles, lactate biosensor, continuous monitoring, broad-range detection, clinical diagnosis

## Abstract

Recycling and revalorization of waste are currently essential for sustainable growth. Mill scale, a waste product from steel production industries, which contains high levels of iron and minimal impurities, is proposed in this study as the source to synthesize magnetite nanoparticles (Fe_3_O_4_) for an enhancement of a lactate biosensor range. The synthesized Fe_3_O_4_ nanoparticles were coated with polydopamine (PDA) to prevent aggregation and degradation, creating a stable platform for immobilizing lactate oxidase enzyme (LOx) on their surfaces. The characterization of the Fe_3_O_4_@PDA material was carried out using transmission electron microscopy (TEM), dynamic light scattering (DLS), and measurement of the polydispersity index (PdI). The Fe_3_O_4_@PDA-LOx material was then deposited on a screen-printed carbon electrode modified with Prussian blue (SPCE-PB) for lactate detection. The biosensor exhibited a broad, dual linear concentration-response range, one from 0.1 to 4.62 mM with a limit of detection of 0.32 mM and sensitivity of 1.54 $\mu A{mM}^{-1}{cm}^{-2}$, and another one from 4.62 to 149.21 mM with a limit of detection of 6.31 mM and sensitivity of 0.08 $\mu A{mM}^{-1}{cm}^{-2}$. The dual-range concentration response of the biosensor makes it an ideal tool for lactate determination in various applications, including sports medicine, clinical diagnosis, and industrial bioprocessing.

## 1. Introduction

Steel is an essential material in our society; however, the steel industry is among the three largest producers of carbon dioxide, a greenhouse gas that contributes to global warming. The United Nations Climate Change Conference (COP21) held in Paris in December 2021 [1] reached a significant agreement to substantially reduce global greenhouse emissions. To address this issue, the European Union (EU) has committed to reducing greenhouse gas emissions by at least 40% by 2030, compared to 1990 levels, under its broader 2030 climate and energy framework. This commitment is part of the EU’s initial nationally determined contribution (NDC) under the Paris Agreement. Reducing greenhouse gas emissions is crucial for mitigating the effects of climate change and preserving the planet for future generations. While the steel industry is an important contributor to our economy, it is essential to reduce carbon footprints and promote sustainable practices.

Therefore, steel-making companies cope with strong pressure to reduce CO_2_ emission from this process plus the amount of residues that they produce. In the steel-making process, due to high temperatures, the outer skin of products oxidizes to iron oxide. This skin that can be easily removed is called mill scale. Mill scale is one of the waste materials that is produced as a result of the hot rolling of steel in all steel companies. If not revalorized, this waste ends up in landfills, requiring an enormous cost due to transportation and the associated CO_2_ emission. The waste can also cause the leaching of some fractions of heavy metals, and present a continuous need to devote more land to landfills. Therefore, it is crucial for steel-making companies to find ways to reuse and recycle mill scale. This can include reprocessing it back into the steel-making process or using it as a raw material for other industries.

Initially, steps have been taken to transform the waste into a valuable product, for instance, through a carbothermic reduction into pure iron [2], or as a valuable additive in cement mortars [3] or concrete [4,5,6]. Moreover, researchers have proposed using mill scale in other applications with more added value. Mill scale has a high content of iron and very low impurities. This makes it a good candidate for the production of iron oxide pigments. Pigments are used in a wide variety of applications such as coating and construction materials [7,8,9,10,11,12]. During the manufacturing process of pigments, the mill scale is resized to nanosized particles. These nanoparticles present valuable properties that are now considered to be of extreme interest due to their physicochemical properties. For instance, Shahid et al. [13,14] used a coprecipitation method to obtain magnetite (Fe_3_O_4_) from the mill scale. The obtained nanoparticles are valued as an Arsenic absorbent from water due to the high surface area, which contributes to a greater absorption power, and Predescu et al. [15] have recently introduced new methodologies to obtain superparamagnetic nanoparticles. The properties of iron oxide nanoparticles, not only obtained from mill scale, are valorized in many fields (i.e., magnetic resonance imaging (MRI), bio-separation, purification, and biosensors) [16,17,18,19,20] due to their superparamagnetic properties at the nanoscale. Besides magnetic characteristics, recently, Gao et al. [21] discovered that intrinsic enzymatic properties could be assigned to the iron oxide nanoparticles broadening the range of applications, especially in the biosensor’s development [22,23,24] or in the enhancement of biogas production [25].

In the field of biosensors, the utilization of nanoparticles with their expansive surface areas has proven instrumental in elevating the overall performance of these critical analytical tools. This enhanced performance is achieved through a twofold approach. Firstly, by capitalizing on the large surface area of nanoparticles, researchers are able to effectively immobilize a great number of bioreceptors, such as antibodies or enzymes, on the sensor surface. This amplifies the sensor’s sensitivity and ability to precisely detect even the most minuscule concentrations of target molecules, ultimately pushing the limits of detection to new heights [26,27]. Moreover, the utilization of nanoparticles extends the range of measurement capabilities, allowing biosensors to tackle a broader spectrum of analyte concentrations [28]. The ability of biosensors to provide accurate measurements across this wide concentration range has broadened their applications across diverse fields, from medical diagnostics to environmental monitoring. Recently, the COVID pandemic has emphasized the need to move to a more sustainable medicine, and the empowerment of the patient is now considered a fundamental element of the follow-up procedure in many illnesses. Therefore, the production of biosensors in a more sustainable way and noninvasive monitoring, for instance, sweat monitoring of different metabolites such as glucose [29,30], cortisol [31], ions [32,33], or lactate [34], has become increasingly important.

Lactate is one of these metabolites in which the expected concentration in sweat compared to blood is extremely different and while the lactate sensors for blood are expected to have high sensitivity, in sweat the requirement is to extend the range. Researchers are focused on expanding the range of measurement while having a feasible wearable device. Garcia et al. [35] already proposed a wide-range lactate sweat sensor based on lactate dehydrogenase to measure lactate, which requires a higher potential for measurements but the system embedded a miniaturized fuel cell to generate the required power. Imani et al. [36] presented a wearable sensing device for lactate monitoring based on a working electrode functionalized with a biocompatible biocatalytic layer (lactate oxidase (LOx)-modified Prussian blue), which enables a range of measurement up to 25 mM. Poletti et al. [37] drop-casted a layer of Nafion on top of the functionalization of the sensor and a flux of sample is created on top of the electrode to enhance the range of lactate monitoring up to 50 mM. Pribil et al. [38] proposed a lactate biosensor based on Prussian blue that can continuously detect lactate concentrations up to 70 mM. Xuan et al. [39] proposed a new strategy for monitoring sweat lactate while also preserving the biosensor response to changes in pH and temperature by incorporating an outer layer composed of polymer, plasticizer, and lipophilic salt onto the electrode surface. Even though these examples enhanced the range of measurement of the state-of-the-art blood-designed lactate biosensors, which is around 1 mM [40,41,42], none of them used green manufacturing processes and most of them used complex and non-environmentally friendly materials, such as carbon nanotubes [43], gold nanoparticles [44], or polymers [45].

In this article, we propose a method to valorize mill scale in a high-added-value application such as the level of lactate monitoring, which is a relevant parameter not only in medical analysis or physical condition monitoring but also in the food industry, since lactate level is correlated with the fermentation level and is an indication of freshness. This paper presents a strategy to obtain iron oxide nanoparticles, which reduces the environmental impact compared to conventional methods that use iron salts as precursors. Furthermore, the utilization of iron oxide NPs in biosensor development allows the sensor to work in an extended range, expanding the use of lactate sensors to new growing fields such as wearable sweat sensing. The sensor’s sensitivity and limit of detection (LOD) were characterized, and its performance was compared with and without nanoparticle incorporation. To the best of our knowledge, this is the first sensor that incorporates mill-scale-produced iron oxide nanoparticles to develop lactate oxidase-based biosensors and achieves a working range exceeding 100 mM of lactate.

## 2. Materials and Methods

### 2.1. Materials and Chemicals

Mill scale from CELSA Group (Castellbisbal, Spain) as a source of iron oxide, dopamine hydrochloride, phosphate buffer saline (PBS) (10 mM, pH 7.4), citric buffer (10 mM, pH 5.5), TRIS buffer solution (10 mM, pH 8.5), lactate oxidase (LOx) from Sorachim (Lausanne, Switzerland), sodium hydroxide (NaOH 2M), potassium chloride (KCl 3M), DI water, and hydrochloric acid (HCl). Biosensor systems were constructed based on a screen-printed Prussian blue/carbon electrode (SPCE/PB) purchased from Metrohm DropSens (Oviedo, Spain). The working electrode consisted of carbon/Prussian Blue (4 mm in diameter), while Ag/AgCl and the carbon ring were the reference and counter electrodes, respectively.

### 2.2. Synthesis of Fe_3_O_4_ Nanoparticles

The synthesis of magnetite nanoparticles relied on the chemical co-precipitation technique. A total of 1 g of mill scale was dissolved in 20 mL of hydrochloric acid and stirred for 48 h on a magnetic stirrer. The obtained orange solution was diluted in 40 mL of deionized water. After that, 50 mL of sodium hydroxide (2M) was added dropwise until the mixture reached a pH of 10 to 11. The mixture was stirred under consistent temperature conditions, and the synthesis was carried out in a nitrogen gas atmosphere to minimize oxygen levels in the system. After 30 min, the system was cooled to room temperature, and the magnetite precipitates were isolated and thoroughly washed from the solution using a magnet. Lastly, the resulting black powder was dried at room temperature.

### 2.3. Synthesis of Fe_3_O_4_@PDA Nanoparticles

To form the Fe_3_O_4_@PDA system, 10 mg of magnetite nanoparticles obtained from the previous step were mixed with 20 mL of TRIS buffer solution (10 mM, pH 8.5). The solution was sonicated to homogeneity. Then, 10 mg of dopamine hydrochloride was added, and the resulting solution was stirred for 24 h at room temperature on a magnetic stirrer while being saturated with O_2_. After 24 h, the Fe_3_O_4_@PDA nanoparticles were collected by magnetic decantation and washed with deionized water to remove any unreacted dopamine. The resulting black powder was allowed to dry for 24 h at room temperature.

### 2.4. Lactate Oxidase Immobilization on the Fe_3_O_4_@PDA Nanoparticles

Lactate oxidase was immobilized on the surface of Fe_3_O_4_@PDA nanoparticles using an adsorption process, by adding 5 mg of the nanomaterial and 1 mg of lactate oxidase to 1 mL of citric buffer (10 mM, pH 5.5). The newly obtained material was centrifuged at 15,000 rpm for 5 min to homogeneity. The immobilization of the material was performed for an optimum time of 24 h at ambient temperature (25 °C, 50% RH). Once finished, the material was magnetically decanted and washed with deionized water to remove any unreacted excess enzyme molecules.

### 2.5. Fabrication of SPCE/Fe_3_O_4_@PDA-LOx Electrode

To fabricate the lactate biosensor system, 2 µL of the obtained Fe_3_O_4_@PDA-LOx nanomaterial (6.5 $mg{mL}^{-1}$) was deposited on the surface of the working electrode modified with Prussian blue and allowed to dry at room temperature. The electrode surface was then rinsed with deionized water to eliminate any excess or unbound enzyme molecules that may have remained on the electrode surface. The biosensor system was stored in a refrigerator at 4 °C when not in use.

**Figure 1:**
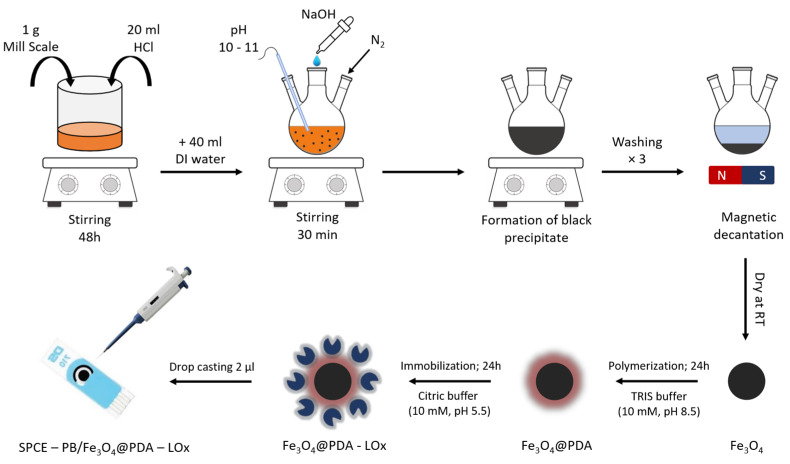
Illustration depicting the step-by-step process involved in the preparation of the lactate biosensor.

To this end, the synthesis and polymerization of the Fe_3_O_4_@PDA material, immobilization process, and electrode modification are illustrated schematically in Figure 1.

### 2.6. Physicochemical Analysis

Transmission electron microscopy (TEM) analysis was carried out using a Joel analyzer (JEM-J1010) with a resolution of 0.45 nm and a maximum acceleration of 100 kV. To assess the stability of the materials in a liquid solvent, zeta potential (ZP) and polydispersity index (PdI) values were determined using a Zetasizer Nano ZS (Malvern Instruments Ltd., Malvern, UK) with a range of 0.6–6000 nm.

### 2.7. Electrochemical Study

Electrochemical measurements were performed using the potentiostat (PalmSens, Houten, The Netherlands), and tests were carried out with the dedicated PSTrace application. For the measurements, the biosensor was immersed in 50 mL of the buffer solution. Cyclic voltammetry (CV) was used to characterize the electrodes. To optimize the electrochemical conductivity, CV experiments were carried out in a mixture of 10 mM PBS (pH 7.4) containing 3M KCl over the relevant potential range of −0.2 and +0.6 V with a scan rate of 20 $mVs^{-1}$. Accordingly, chronoamperometry (CA) was used to assess the lactate response. CA experiments were conducted at a constant potential of −0.15 V by dissolving various concentrations of lactate in a stirred solution of PBS (10 mM, pH 7.4). The background current was allowed to stabilize for 200 s, and sequential injections of lactate were made at 100 s intervals. The current after each lactate addition was measured and used for the lactate calibration curves. All measurements were conducted under ambient temperature conditions (25 °C, 50% RH).

## 3. Results and Discussion

### 3.1. Morphological Characterization of Fe_3_O_4_ and Fe_3_O_4_@PDA

Magnetite nanoparticles were coated with a polymeric layer, polydopamine, to mitigate challenges such as aggregation and degradation that arise when using magnetite nanoparticles in biosensor applications. Dopamine, which draws inspiration from mussel adhesive proteins, has gained significant attention due to its biocompatibility and adhesive characteristics, attributed to its two phenolic hydroxyl groups that form stable complexes with various molecules and materials, including magnetite nanoparticles. Moreover, dopamine can establish –COO–H_3_N– ion pairs through interaction with the carboxyl groups on the Fe_3_O_4_ surface and, notably, it can undergo polymerization under basic conditions, facilitating the creation of a well-defined polymeric shell [46,47,48].

**Figure 2:**
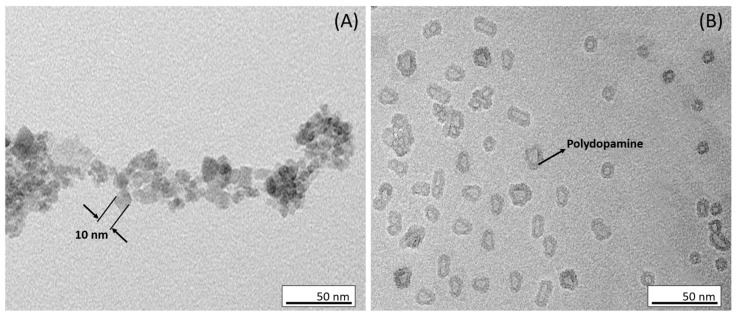
Transmission electron microscopy (TEM) of (**A**) Fe_3_O_4_, and (**B**) Fe_3_O_4_@PDA.

Transmission electron microscopy (TEM) was employed to investigate the morphological structure of Fe_3_O_4_ and Fe_3_O_4_@PDA. As depicted in Figure 2A, the magnetite nanoparticles exhibited a consistently spherical morphology with diameters in the range of ≈10 nm. The narrow size distribution of the nanoparticles indicates that they were well synthesized and are suitable for further functionalization.

The second TEM image (Figure 2B) illustrates the Fe_3_O_4_ nanoparticles after being coated with a polydopamine layer. The magnetite nanoparticles appear to be surrounded by a PDA layer unevenly, creating a matrix of an irregular shape. This observation indicates that the formation process of the Fe_3_O_4_@PDA system was successful.

**Figure 3:**
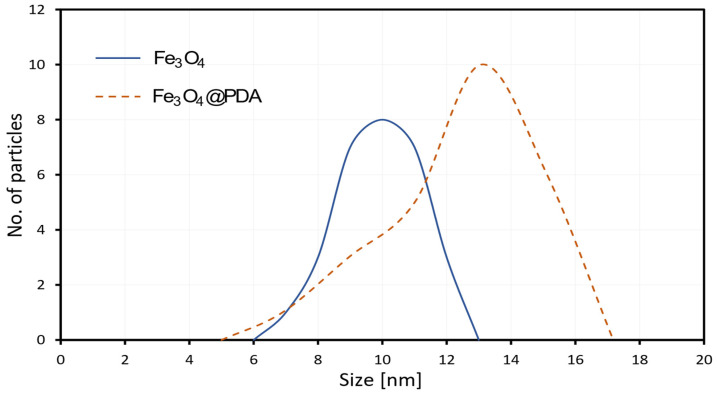
A particle size distribution histogram determined from the TEM images.

Figure 3 shows a particle size distribution histogram determined from the TEM images showing the variation in the particle size for both nanoparticles with and without polydopamine coating.

### 3.2. Colloidal Stability and Evaluation of Particle Size

Electrokinetic analysis was performed at a constant pH of 7.0 to determine the stability of Fe_3_O_4_ and Fe_3_O_4_@PDA nanoparticles. To evaluate the effectiveness of the formation of the Fe_3_O_4_@PDA system and its components, the zeta potential was determined (see Table 1). The calculated zeta potential of (−29.8 mV) for Fe_3_O_4_ nanoparticles demonstrates the significant stability of magnetite colloid within the solution. Following surface modifications, there was a reduction in zeta potential to −17.3 mV for Fe_3_O_4_@PDA, implying that these modifications led to decreased electrostatic repulsions between the nanoparticles. Nevertheless, it is worth noting that the obtained values of zeta potential, although lower, still indicate a moderate level of stability for the materials in the studied solution.

Particle size analysis was conducted for the investigated system, and the outcomes are presented in Table 1. When comparing them to bare magnetite nanoparticles, the presence of a PDA coating significantly influences the hydrodynamic particle size. The successive increase in the particle sizes across the materials indicates the successful execution of the synthesis processes. The variation in size measurements acquired through dynamic light scattering (DLS) and transmission electron microscopy (TEM) can be attributed to the tendency of the material to agglomerate. Furthermore, the polydispersity index was calculated using dynamic light scattering (DLS) to evaluate the uniformity of particle dispersion. Results show that the index was found to be 0.388 and 0.469 for Fe_3_O_4_ and Fe_3_O_4_@PDA, respectively.

### 3.3. Electrochemical Study of SPCE-PB and SPCE-PB/Fe_3_O_4_@PDA-LOx-Modified Electrodes

**Figure 4:**
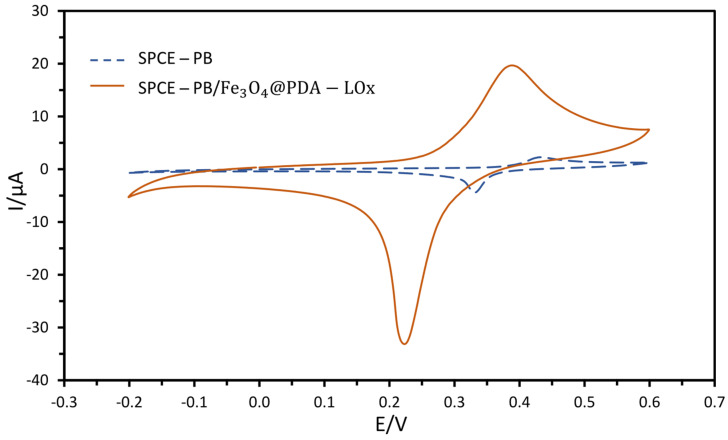
Cyclic voltammetry diagram for SPCE-PB and SPCE-PB/Fe_3_O_4_@PDA-LOx electrodes in PBS (10 mM, pH 7.4) containing 3M KCl at 20 mV s^−1^ scan rate.

For electrochemical characterization of the analyzed system, cyclic voltammograms for the unmodified SPCE-PB and modified SPCE-PB/Fe_3_O_4_@PDA-LOx electrodes were investigated in PBS (10 mM, pH 7.4) containing 3M KCl (Figure 4). The results revealed a peak-to-peak separation of 101 and 161 mV for SPCE-PB and SPCE-PB/Fe_3_O_4_@PDA-LOx electrodes, respectively. These values illustrate the irreversible behavior of [Fe(CN)_6_]^3−/4−^ at the electrodes under investigation. The deposition of the Fe_3_O_4_@PDA-LOx material on the surface of SPCE-PB results in a significant increase in oxidation/reduction peaks. Moreover, the redox peak current increased from −3.16 µA for the SPCE-PB electrode to −22.17 µA for the modified SPCE-PB/Fe_3_O_4_@PDA-LOx electrode, suggesting a higher electroactive surface area. The obtained results are in good agreement with the results reported in the literature, indicating the fast electron transfer behavior of magnetite nanoparticle-modified electrodes [49,50].

**Figure 5:**
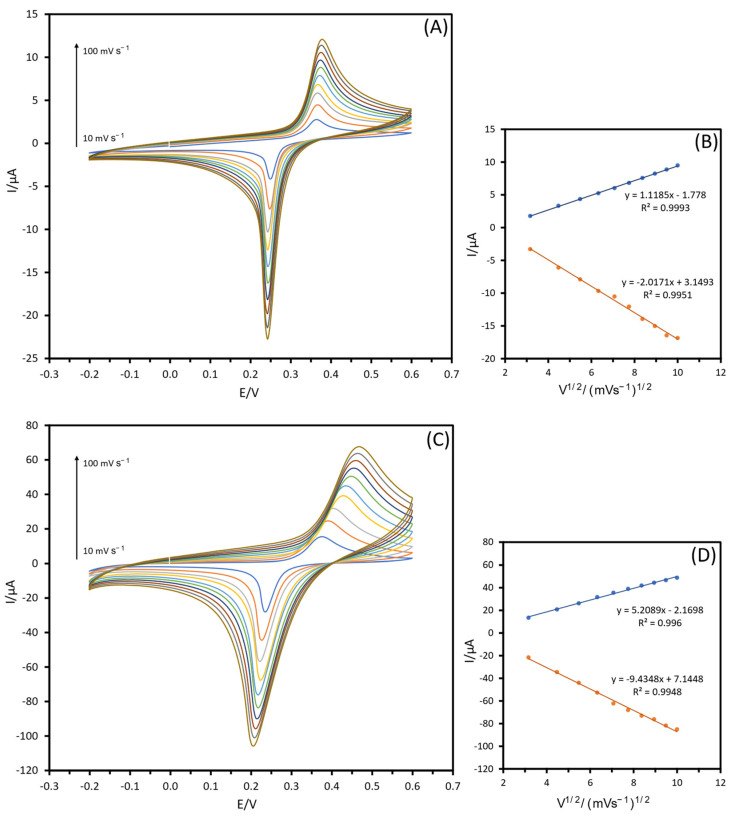
Cyclic voltammograms of (**A**) SPCE-PB, and (**C**) SPCE-PB/Fe_3_O_4_@PDA-LOx in PBS (10 mM, pH 7.4) in presence of 3M KCl at different scan rates (in the range from 10 to 100 mV s^−1^); (**B**,**D**) calibration plots of peak current vs. square root of the scan rate.

Next, a voltammetric scan rate study was used to test the electrochemical activity of both unmodified and modified electrodes. Figure 5 shows the CVs of SPCE-PB and SPCE-PB/Fe_3_O_4_@PDA-LOx electrodes in PBS (10 mM, pH 7.4) containing 3M KCl at various scan rates (10–100 mV s^−1^). As shown in Figure 5A, the CV of the unmodified SPCE-PB electrode exhibits a pair of well-defined peaks at E^red^ = 0.24 V, which can be ascribed to the redox transition of PB. The effect of various scan rates on the electrochemical properties of the SPCE-PB/Fe_3_O_4_@PDA-LOx electrode is depicted in Figure 5C. The redox current exhibited a rise with the increasing scan rate, ranging from 10 to 100 mV s^−1^. The shape of the CV indicates that there are no other additional redox processes, confirming a single-electron transition. The relationship between current ($I_{p}$) and the square root of the scan rate (V^1/2^) (Figure 5B,D) serves as an important diagnostic criterion when employing cyclic voltammetry to determine the nature of the reaction mechanism. The results firmly suggest that the redox reaction of LOx on the electrode surface conforms to a quasi-reversible diffusion-controlled electrochemical operation.

The response of SPCE-PB/ Fe_3_O_4_@PDA-LOx to lactate can be described by Equations (1)–(3):

(1) $$Lactate+O_{2}\overset{LOx}{\to }Pyruvate+H_{2}O_{2}$$

(2) $$H_{2}O_{2}+{PB}_{red}\to {PB}_{ox}+{OH}^{-}$$

(3) $${PB}_{ox}\to {PB}_{red}+e^{-}$$

**Figure 6:**
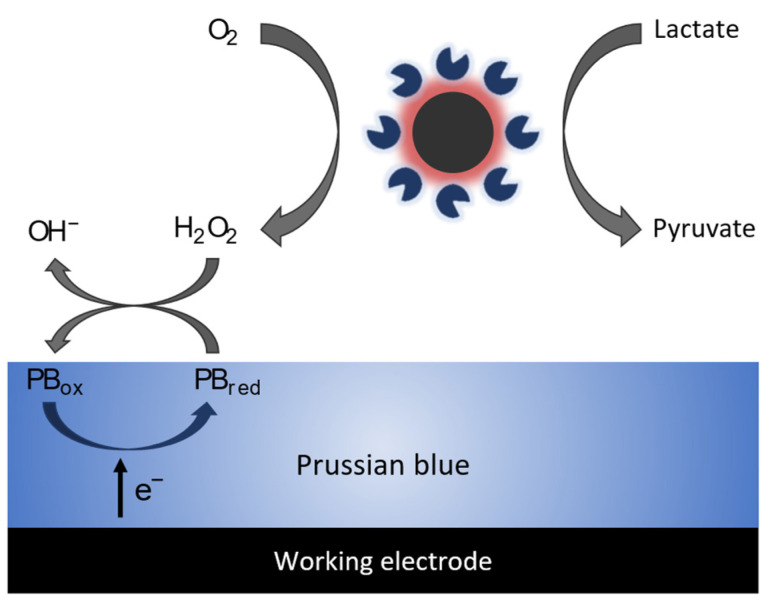
Schematic of the enzymatic reactions on the surface of the working electrode.

Lactate was oxidized by LOx to generate pyruvate and hydrogen peroxide (H_2_O_2_) as the subproduct, which is then detected through Prussian blue (PB) as the redox mediator. Firstly, the PB layer is activated at a constant potential and is then electrochemically reduced from its original oxidized state (PB_ox_) to reduction (PB_red_). Following this, the PB reduction is subsequently oxidized back to PB oxidize in the presence of hydrogen peroxide, leading to detection. The oxidation of the mediator releases an electron that can be detected by the electrode, generating an electrochemical signal that is proportional to the lactate concentration. Figure 6 illustrates the enzymatic reactions taking place at the surface of the working electrode developed in this work.

**Figure 7:**
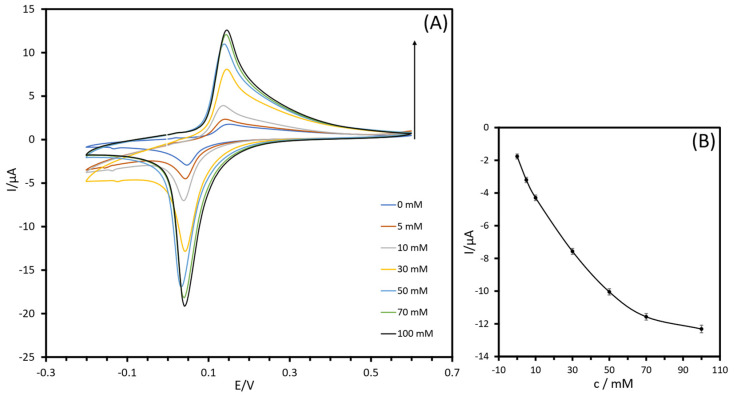
(**A**) Cyclic voltammograms of SPCE-PB/Fe_3_O_4_@PDA-LOx electrode in PBS (10 mM, pH 7.4) for different concentrations of lactate at scan rate of 20 mV s^−1^; (**B**) calibration graph (n = 3).

Electrochemical investigations were performed to characterize the response of the SPCE-PB/Fe_3_O_4_@PDA-LOx electrode. Cyclic voltammograms (CVs) were obtained by immersing the sensor in a 10 mM phosphate buffer solution (pH 7.4) without lactate and in the presence of lactate, ranging from −0.2 V to +0.6 V, at a scan rate of 20 mV s^−1^. Upon addition of lactic acid (to a final concentration of 100 mM), there was an enhancement of the anodic peak current concomitant with a decrease in the cathodic peak current (Figure 7A).

The correlation between peak current and lactate concentration is presented in Figure 7B. The curve illustrates that the voltammetric response approached saturation as the lactate concentration exceeded 100 mM. The current issue is that the production of hydrogen peroxide by the oxidase has reached a point where it is inhibiting the enzymatic activity of LOx. Despite the reduced amount, the quantity obtained is still sufficient for continuous monitoring of lactate concentrations over an extended period, particularly in cases where lactate levels are high. To tune the linear range, improvements can be made in the fabrication of the lactate sensor. This could involve using different polymeric membranes and/or stabilizing agents to regulate the diffusion of the analyte through the biosensor, which can enhance the operational stability of the enzyme [51]. Freeman et al. [52] proposed the addition of cellulose acetate on the surface of the working electrode, creating a diffusion-limited layer. While this layer effectively slowed the diffusion and extended the sensor’s linear range to higher concentrations, the sensor was only capable of detecting lactate concentrations up to 30 mM, and the resulting reduction in current density limited its overall sensitivity.

**Figure 8:**
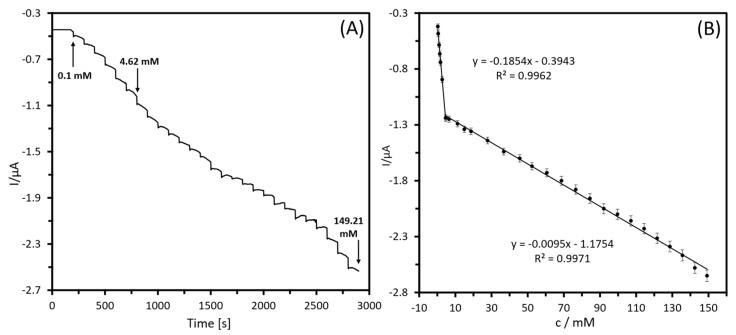
(**A**) Chronoamperometric response of SPCE-PB/Fe_3_O_4_@PDA-LOx electrode in PBS (10 mM, pH 7.4) on injection of various concentrations of lactate at a working potential of −0.15 V; (**B**) the resulting calibration curve (n = 3).

Following an adjustment of the settings, the biosensing system’s analytical response was analyzed through chronoamperometry, in which varying amounts of lactate were sequentially injected and continuously stirred into PBS (pH 7.4). An optimum applied potential of −0.15 V was chosen based on the onset potential for electro-oxidation of lactate by the fabricated biosensor, obtained during cyclic voltammetry studies to run the amperometry experiment since this potential produced a better change in the measured signal and, therefore, more sensitivity of response. Also, this low applied potential could reduce interfering signals from other electroactive species in the sample, increase sensitivity, and produce a longer electrode lifespan. The analytical performance of the prepared Fe_3_O_4_@PDA-LOx biosensor was assessed by evaluating its response to lactate concentrations varying from 0.1 to 149.21 mM (Figure 8A). It is observed that with increasing lactate concentration the current changed, indicating good activity with efficient mass transport and fast electron transfer properties. According to the corresponding calibration plot, see Figure 8B, the sensor response displayed two linear concentration ranges: one from 0.1 to 4.62 mM with a correlation coefficient of 0.9962 and sensitivity of 1.54 $\mu A{mM}^{-1}{cm}^{-2}$, and another from 4.62 to 149.21 mM with a correlation coefficient of 0.9971 and sensitivity of 0.08 $\mu A{mM}^{-1}{cm}^{-2}$. The presence of two linear ranges in the lactate response of the biosensor can be explained by the interaction between the increased analyte concentration and the limited amount of LOx present in the biosensor. When the lactate concentration is low, there are sufficient active enzyme sites available for rapid substrate conversion, leading to a strong and linear current response. However, as the lactate concentration increases, the enzyme becomes saturated and unable to keep up with the rate of substrate conversion, resulting in a decrease in the number of active enzyme sites. This leads to a decrease in the current response and slower reaction kinetics, which in turn results in a decrease in sensitivity towards higher lactate concentrations [53]. Shitanda et al. [54] developed a screen-printed sensor by employing grafted MgO-templated carbon (GMgOC) as the working electrode material to achieve stable immobilization of enzymes containing amino groups and mediators. Nonetheless, the outcomes of their study validated the correlation between lactate concentrations ranging from 1 to 50 mM by applying a potential of +0.1 V.

The linear regression parameters that were obtained from correlating the lactate concentration with the analytical signal for catalysis were utilized to determine the limit of detection (LOD). The limit of detection of the SPCE-PB/Fe_3_O_4_@PDA-LOx electrode was calculated as 0.32 mM for lactate concentrations ranging from 0.1 to 4.62 mM, and 6.31 mM for concentrations ranging from 4.62 to 149.21 mM, using the following equation:

(4) LOD=3×SDm

where SD is the standard deviation of the current value, and m is the slope of the calibration curve [55].

From an analytical standpoint, lactate concentration in sweat is approximately 10 times higher than in blood. During a progressive, incremental exercise test, blood lactate concentration typically ranges from 8 to 10 mM, while during intense exercise it can increase to 15–25 mM [56]. Contrarily, in sweat, the lactate concentration can increase from 10 mM to over 100 mM with increased exercise intensity [57]. The dual linear concentration-response ranges observed in the sensor make it highly versatile and suitable for lactate determination in a wide range of sample types, regardless of their lactate content. The lower concentration range, spanning from 0.1 to 4.62 mM, enables the detection of low levels of lactate, making it invaluable for samples with potentially low lactate concentrations, such as biological fluids: blood, sweat, urine, and cell cultures for lactate production monitoring. Conversely, the higher concentration range of 4.62 to 149.21 mM is advantageous for samples with high lactate content, such as those found in muscle tissue during intense exercise or in fermentation processes. This range ensures accurate and precise quantification of lactate concentration, even at high levels.

The performance of the lactate biosensor proposed in this study was evaluated and compared with similar devices reported by the scientific literature (Table 2). However, due to the significant differences in the manufacturing processes, it is challenging to make a direct comparison between the biosensors.

Nonetheless, the biosensor developed in this study demonstrated exceptional sensitivity across a broad linear range, utilizing a low potential value. The excellent performance of the biosensor can be attributed to the utilization of magnetite nanoparticles synthesized from raw mill scale. These nanoparticles exhibited unique physicochemical properties, which contributed to the sensitivity and specificity of the biosensor. The promising results obtained from this study suggest that synthesized magnetite nanoparticles are an excellent candidate for monitoring lactate levels in a wide range. To end this, the biosensor developed in this study can provide a low-cost and efficient method for monitoring lactate levels and the use of waste materials, such as mill scale, in the synthesis of nanoparticles to reduce the environmental impact of nanoparticle production.

## 4. Conclusions

In this work, we have presented a new and innovative method to valorize mill scale in a high-added-value application such as the enhancement of a biosensor for lactate level monitoring. It runs at a very low applied potential (−0.15 V), which minimizes interfering signals from other electroactive species and increases sensitivity, leading to more reliable and efficient lactate sensing. The biosensor showed the ability to detect lactate in two distinct linear concentration ranges, one from 0.1 to 4.62 mM, with a limit of detection of 0.32 mM, and another one from 4.62 to 149.21 mM with a limit of detection of 6.31 mM, a range that expanded the current state-of-the-art electrochemical biosensors by 50% and enables the use of these sensors in such wearable applications for lactate monitoring in sweat. The proposed lactate oxidase-based biosensor exhibited good sensitivity in both concentration ranges, with a value of 1.54 $\mu A{mM}^{-1}{cm}^{-2}$ for the lower range, and 0.08 $\mu A{mM}^{-1}{cm}^{-2}$ for the higher range. This dual-range concentration response of the biosensor makes it a valuable tool for lactate determination in various applications, including sports medicine, clinical diagnosis, and industrial bioprocessing. The novel method introduced in this study represents a significant advancement in sustainable and environmentally friendly approaches to biosensor development. However, various analyses can be conducted to enhance the optimization of the sensor. These may include assessing the sensor’s performance with real samples, implementing continuous monitoring applications for point-of-care measurements, and exploring utility in the food industry.

## Figures and Tables

**Table 1:** Zeta potential, hydrodynamic particle diameter, and polydispersity index (PdI) values for Fe_3_O_4_ and Fe_3_O_4_@PDA systems.

Sample	Zeta Potential (mV)	Average Size (nm)	Polydispersity Index (PdI)
Fe_3_O_4_	−29.8	192.2	0.388
Fe_3_O_4_@PDA	−17.3	463.7	0.469

**Table 2:** Comparison of analytic performance for lactate detection through amperometric techniques.

Electrode Modification	Solvent	Linear Range (mM)	Sensitivity $\left(\mu A{mM}^{-1}{cm}^{-2}\right)$	Potential Studied (V)	Ref.
SPCE-PB/Fe_3_O_4_@PDA-LOx	PBS (pH 7.4)	0.1–4.62 4.62–149.21	1.54 0.08	−0.15	This work
SPE-PB/LOx + GO-Ch	Artificial sweat (pH 4.7)	1–50	0.39	+0.0	[37]
SPE/GA-LDH/AuNPs-ERGO-PAH	PBS (pH 7.5)	0.5–3	1.08	+0.5	[40]
Printed AgNPs/BSA-LOx	PBS (pH 7.4)	1–20	0.26	+0.65	[58]
SPCE/Graphene/PB/PVA-SbQ-LOx	PBS (pH 7.4)	0.25–5	1.64	−0.1	[59]
Au/CNT/b2LOxS/PEI-PEGDGE/CA	Artificial sweat (pH 5.4)	0.5–20	0.41	+0.15	[60]

Acronyms: SPCE: screen-printed carbon electrode; PB: Prussian blue; PDA: polydopamine; LOx: lactate oxidase; GO: graphene oxide; Ch: chitosan; GA: glutaraldehyde; LDH: lactate dehydrogenase; NPs: nanoparticles; PAH: poly(allylamine) hydrochloride; BSA: bovine serum albumin; PVA-SbQ: poly(vinyl alcohol)-bearing styryl pyridinium groups; CNT: carbon nanotubes; b2LOxS: DET-type engineered LOx; PEI: polyethylenimine; PEGDGE: poly(ethylene glycol) diglycidyl ether; CA: cellulose acetate.

## Data Availability

Not applicable.
